# Life satisfaction and musculoskeletal complaints in a population seeking osteopathy care: consecutive sample of 611 patients

**DOI:** 10.1186/s12998-020-00303-y

**Published:** 2020-03-11

**Authors:** Brett Vaughan, Jane Mulcahy, Thomas Allen, Emi Coupe, David Gobbo, Leila Nasser, Karen Pain, Kylie Fitzgerald

**Affiliations:** 1 Department of Medical Education, University of Melbourne, Melbourne, Australia; 2 College of Health & Biomedicine, Victoria University, Melbourne, Australia; 3 Independent Researcher, Melbourne, Australia

**Keywords:** Patient report outcome measure, Osteopathic medicine, Satisfaction with life

## Abstract

**Background:**

Life satisfaction is a component of the subjective well-being construct. Research consistently suggests that life satisfaction is associated with enhanced social benefits and improved health outcomes. However, its relationship to musculoskeletal health outcomes is underexplored. This study evaluates the life satisfaction of a patient population presenting with musculoskeletal complaints, and the relationship of life satisfaction with other health demographics and behaviours.

**Method:**

The study used a consecutive sampling design. Patients attending the Victoria University Osteopathy Clinic (Melbourne, Australia) were invited to complete the PROMIS® General Life Satisfaction scale (GLSS) along with questions related to health demographics and behaviours.

**Results:**

The GLSS T-score was not significantly different for gender, being born outside of Australia, speaking English at home, or complaint chronicity.

**Conclusions:**

Life satisfaction did not appear to be related to a range of health and demographic variables in the current musculoskeletal pain cohort. The PROMIS® General Life Satisfaction scale could prove useful to explore the relationship between life satisfaction and treatment outcomes for musculoskeletal complaints.

## Background

Satisfaction with life (SWL) is one of the components of subjective well-being (SWB) [1] and relates to the subjective cognitions and judgments we make about our lives [2]. The other component is the affective, or the positive and negative emotions that constitute affect. People form judgments of how satisfied they are based on their perception of emotional experiences, with the number of positive experiences having a greater impact on higher ratings of SWL than negative emotions [3]. Higher SWL has also been associated with lower levels of morbidity [4], mortality [5] and improved psychological health [6].

There is a volume of literature that describes the impact of life satisfaction across a range of health behaviours. For example, Grant et al. [7] reported that avoiding cigarette smoking and dietary fat, and increasing physical exercise, use of sunscreen, and fruit intake were positively associated with increased life satisfaction in a group of 17–30 year olds, with the exception of alcohol consumption and fiber intake. Physical activity and non-smoking were most significantly associated with higher life satisfaction, across all countries in this work. Similar results were demonstrated by Baumann et al. [8] in their longitudinal study spanning 5 years, assessing life satisfaction and cardiovascular risk factors such as: physical inactivity; smoking; obesity and hypercholesterolemia, in a patient group with coronary artery disease. Both studies [7, 8] highlighted a strong link between low SWL and physical inactivity, but acknowledge the probability that physical activity and SWL is bidirectional.

In the context of musculoskeletal practice, researchers and clinicians have explored the relationship of SWL of patients presenting with a musculoskeletal conditions with pain severity. Talei-Khoei et al. [9] concluded that high SWL had a buffering effect on pain and reduced pain catastrophisation in patients suffering from upper limb musculoskeletal conditions. Boonstra et al. [10] compared SWL between a healthy population (no symptoms of pain or no presenting condition?) with those suffering from chronic musculoskeletal pain. The latter population demonstrated lower global SWL and statistically lower satisfaction levels across six of eight satisfaction domains (self-care, leisure, vocational and financial situation, sex life and contacts with friends). In other musculoskeletal pain work, Espi-Lopez et al. [11] demonstrated improvements in SWL with osteopathy care for tension type headaches, however the nature of the association is purely speculative. Together, these studies suggest that life satisfaction should be considered in the management of musculoskeletal complaints, particularly those that are chronic.

Vaughan et al. [12] also demonstrated that lower life satisfaction may be associated with lower levels of health literacy in a population seeking osteopathy care. However, there is limited research into the relationship between health literacy, life satisfaction and outcomes from manual therapy. Other non-musculoskeletal research exploring the relationship of health literacy and life satisfaction is minimal, but suggests associations between these two variables [13]. This health literacy relationship also appears to be the case for other measures of subjective well-being [14] and quality of life [15].

Life satisfaction measures have been well researched with proven psychometric properties and demonstrated validity and reliability [2, 16], the most commonly used being the Satisfaction with Life Scale (SWLS) [2]. This scale has been extensively tested and its reliability, validity and internal consistency is widely accepted. Satisfaction with life data is commonly collected on a large scale to aid in health policy and budget allocations [17], and in clinical settings to understand how a patient perceives their current life. Single-item life satisfaction measures have also been developed and evaluated with studies supporting their reliability [18] and validity [19]. Single item measures may also reduce the administrative burden for patients and clinicians [20]. Lucas & Donnellan investigated single item scales across four large national panel studies and concluded that “… .single-item measures of life satisfaction might be more reliable than some approaches indicate” (p. 330) [18] however their validity may require additional investigation.

The Patient-Reported Outcome Measurement Information System (PROMIS®) Short Form v1.0 - General Life Satisfaction 5a scale (GLSS) represents a more contemporary measure of life satisfaction. The National Institute of Health (NIH) roadmap initiative (www.nihpromis.org) was created to validate, standardize and develop a series of measurements to access patient-reported outcome measures [21]. The PROMIS collection of measures draws upon the three major domains of physical, social and mental health, identified by the World Health Organisation (WHO). To our knowledge the GLSS has not been used to investigate the life satisfaction of patients seeking care for musculoskeletal complaints. We have previously identified that those seeking osteopathy care in the student-led clinic environment report high levels of SWL with a single-item measure [22]. The aim of the current study was to evaluate the relationship of life satisfaction with other health demographics and behaviours, in patients seeking care for musculoskeletal complaints. This data will contribute to our understanding of life satisfaction as a potential factor associated with outcomes of osteopathy care.

## Methods

The study was approved by the Victoria University (Melbourne, Australia) Human Research Ethics Committee (HRE15–035). The study utilised a consecutive sampling design and data analysed in the current work is part of a larger study into the health behaviours, demographics and experiences of patients attending for musculoskeletal care at the clinic [22].

### Participants

Patients presenting for their initial consultation at a student-led osteopathy clinic were invited to participate in the study. Data was collected between January 1 and June 30, 2018. The clinic is located on the Victoria University campus in the Melbourne central business district and is a clinical training environment for osteopathy students completing the final 2 years of their five-year program. All new patients were invited to complete a health demographic and clinical information questionnaire (developed for the larger study [22]) prior to their consultation. New patients who completed a standard patient clinical form (patient identifiers) were also provided with the health demographic and clinical information questionnaire. Patients had an option to ‘opt out’ of the research study by indicating this on their new patient form. Consent to participate was taken if the patient completed the questionnaire and did not select the ‘opt-out’ of the study response on the from. Responses from patients under the age of 18 were excluded.

### Questionnaire

Patients completed a health demographic and clinical information questionnaire. This questionnaire was designed to collect information about a range of health behaviours and current health status, consistent with data collected in the Australian population health surveys [23, 24]. Patients were also invited to complete two measures of life satisfaction: a single life satisfaction question *How satisfied are you with your life?* rated on an anchored Likert-type scale from 0 (not at all satisfied) to 5 representing (extremely satisfied) [25]; and, the GLSS (5 items) rated on a 7-point Likert-type scale from 1 (Strongly disagree) to 7 (strongly agree) in addition to the PROMIS Scale v1.2 - Global Health [26]. Only the items comprising the PROMIS Scale v1.2 - Global Mental 2a 1) *Global04*: In general, how would you rate your mental health, including your mood and your ability to think? 2) *Global05*: In general, how would you rate your satisfaction with your social activities and relationships?), PROMIS Scale v1.2 - Global Physical 2a (*Global03*: In general, how would you rate your physical health? 2) *Global06*: To what extent are you able to carry out your everyday physical activities such as walking, climbing stairs, carrying groceries, or moving a chair?) and Pain Intensity (*Global7 items*) were extracted from the Global Health scale and scored [27].

### Data analysis

Each new patient form and questionnaire was screened and relevant data including: age, gender, postcode, occupation and clinical information was extracted from the clinical history by a single author (BV), then de-identified. Data from each of the forms was entered into SPSS (IBM Corp, USA) for analysis. The GLSS, Global Mental, Global Physical and Pain Intensity scales were scored using the Health Measures Scoring Service (https://www.assessmentcenter.net/ac_scoringservice) and results entered into SPSS.

Descriptive data was generated for each item on the health information questionnaire, the GLSS, Global Mental and Global Physical scales and single-item life satisfaction question. Correlation and inferential statistics were used to evaluate the associations between these items and other data extracted from the health demographic and clinical information questionnaire, and patient clinical history. Alpha was set at *p* < 0.05 and 95% confidence intervals calculated. Effect sizes were also calculated where relevant.

## Results

The present study sampled 632 adults. Of these 212 (33.5%) declined to participate (opted out) or did not complete the health information questionnaire. Nine patients (1.4%) were under the age of 18 years. The available data for analysis was for 411 patients representing a 65% response rate and it is this data presented here. Mean age was 33.5 (±13.2) years, with most patients being female (*n* = 247, 60.1%). Demographic and clinical characteristics for the sample are in Table 1.

**Table 1:** Demographic data for patients participating in the study

Gender	
Male	164 (39.9%)
Female	247 (60.1%)
Age	
Mean (±SD) years	33.47 (±13.2)
Range	19–84 years
Pain Stage	
Acute	183 (44.5%)
Chronic	227 (55.2%)
Region of Presenting Complaint	
Spine & pelvis	238 (57.9%)
Upper extremity	61 (14.8%)
Lower extremity	98 (23.8%)
Born in Australia	
Yes	261 (63.5%)
No	150 (36.5%)
Speak English at Home	
Yes	380 (92.5%)
No	28 (6.8%)
Tobacco Smoking	
Yes	64 (15.6%)
No	288 (70.1%)
VU Osteopathy Student	
Yes	23 (5.6%)
No	388 (94.4%)
Delayed seeing a doctor or health professional due to cost	
Yes	136 (33.1%)
No	242 (58.9%)
Unsure	15 (3.8%)
Delayed buying medicines due to cost	
Yes	58 (14.1%)
No	330 (80.3%)
Unsure	6 (1.5%)

Note: percentages that do not add to 100% represent missing data

**Fig. 1:**
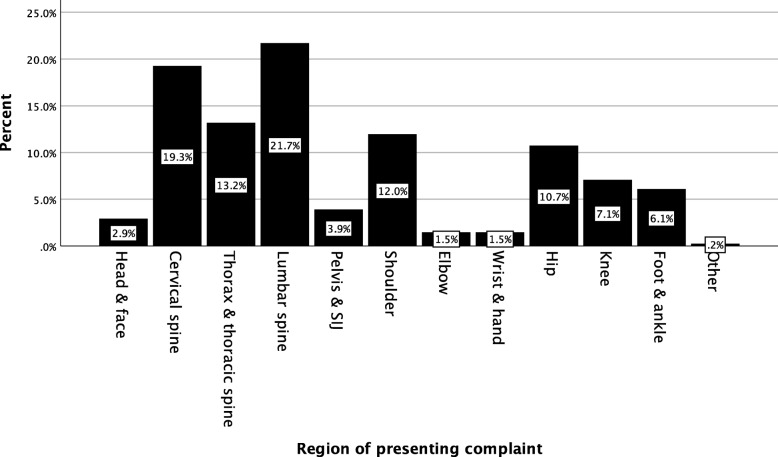
Anatomical region of the primary musculoskeletal presentation (by number of patients)

Anatomical region of the presenting complaint is shown in Fig. 1. Descriptive statistics for the GLSS are presented in Table 2. The single-item life satisfaction question mean was 3.92 (±0.83) and a median of 4 [IQR 4–4]. The Global Health Scale subscale scores are presented in Table 3. The Cronbach’s *alpha* of the GLSS was 0.91 [95%CI 0.90–0.93].

**Table 2:** Descriptive statistics for the General Life Satisfaction scale items and total score

Item	Mean (SD)	Median [IQR]	Range
1. So far I have gotten the important things I want in life	5.52 (1.30)	6 [5–6]	1–7
2. My life situation is excellent	5.53 (1.18)	6 [5–6]	1–7
3. I am satisfied with my life	5.68 (1.22)	6 [5–6]	1–7
4. If I could live my life over, I would change almost nothing	4.90 (1.63)	5 [4–6]	1–7
5. In most ways, my life is close to perfect	4.92 (1.47)	5 [4–6]	1–7
T-score	54.32 (8.90)	55.20 [49.27–58.30]	23.0–73.4

**Table 3:** Additional PROMIS Scales and item descriptive statistics

Item	Mean (SD)	Median [IQR]	Range
Mental Health 2a T-score	50.30 (8.23)	50.80 [44.10–52.70]	25.80–64.60
Physical Health 2a T-score	49.39 (7.51)	50.30 [44.30–56.00]	28.80–63.30
How would you rate your pain on average? (*Global7*)	4.34 (1.99)	4 [3–6]	0–10

### Associations with demographics, health behaviours and pain

The GLSS T-score was not significantly different for gender (*p* = 0.50, 95%CI [− 1.16, 2.36]), being born outside of Australia (*p* = 0.11, 95%CI [− 3.22, 0.35]), speaking English at home (*p* = 0.56, 95%CI [− 2.41, 4.46]), complaint chronicity (*p* = 0.21, 95%CI [− 0.62, 2.85]) or smoking (*p* = 0.27, 95%CI [− 1.03, 3.70]). Age (*r* = − 0.07, 95%CI [− 0.17, 0.02]), PROMIS Physical Health (*r* = 0.07, 95%CI[− 0.03, 0.17]) and PROMIS Mental Health (*r* = 0.04, 95%CI [− 0.05, 0.14]) were not correlated with the GLSS T-score and a trivial correlation was observed for average pain rating (*Global7*) (*r* = 0.01, 95%CI [− 0.08, 0.11]). For other health behaviours, results were analysed for those who indicated *yes* or *no* to a behaviour. Those who reported *delaying buying prescribed medicines* demonstrated higher GLSS T-scores (56.6 ± 10.5 v 54.1 ± 8.3), with the difference being significantly different (*p* = 0.02, 95%CI [0.17, 5.03]). *Delaying seeing a health professional for financial reasons* was also significantly different. Those who reported delaying health visits demonstrated a higher mean GLSS T-score (55.8 ± 7.9 v 53.6 ± 6.0), that was significantly different (*p* = 0.02, 95%CI [0.38, 4.04]).

## Discussion

This study explored life satisfaction in a population presenting with musculoskeletal complaints at a student-led osteopathy clinic. Further, we explored the association of life satisfaction with a range of health behaviours and demographics. In our work, the mean GLSS T-score was 54.32 +/− 8.90, suggesting the average life satisfaction across the cohort is slightly higher than the American general population. T-scores are standardised scores for PROMIS measures - a score of 50 is the American population mean [28] - and there are no Australian data for comparison at present. Establishing an Australian dataset for the GLSS would enable to the comparison of clinical populations in the future.

Previous research has suggested that measurement of overall life satisfaction is independent of gender in adult populations [29], and this also appears to be the case across cultures [30–32]. Conversely, some literature suggests that women have a higher domain life satisfaction when outdoors, and are more satisfied with their family life than men [33] although this is not a consistent finding [34]. The findings of the current work suggest there is no gender difference for overall life satisfaction in those patients presenting to a student-led teaching clinic for care of a musculoskeletal complaint. We did not explore domains of life satisfaction (e.g. job satisfaction, relationship satisfaction, housing satisfaction) and the influence of gender. However, this does provide an avenue for further research.

Literature also suggests there may be cultural differences for life satisfaction. Those born overseas (with respect to the country where the research took place), and those that do not speak English at home tended to have lower overall satisfaction with life [35, 36]. One study looking into immigration to western countries in Europe found that immigrants had significantly lower satisfaction with life [35]. However, those authors explored factors such as socio-economic status and occupation of the participants [35], variables that were not explored within our study. Lafrenière, Sedikides [36] demonstrated the opposite - those from western countries or backgrounds had a lower satisfaction with life than those who migrated from a non-western country, or spoke a languages other than English at home [36]. We explored the effects of country of birth (born/not born in Australia and English language spoken/not spoken at home) and satisfaction with life in our population seeking care for a musculoskeletal complaint. Our results showed that there was no significant difference in satisfaction with life for those born overseas compared to those born in Australia, nor was there a difference for those who speak English at home compared to those who don’t. Socioeconomic status, occupation and work status of the participants were not established for the current study and provide an avenue for further research.

A systematic review by Grant et al. [7] and empirical work by Siahpush et al. [28] suggests a likely positive association between general health status and life satisfaction. In the PROMIS measures, general health status is comprised of physical and mental health domains and, to our knowledge, the current study is the first to explore the general health/life satisfaction relationship using these measures. Physical health has been associated with life satisfaction [34, 37]. In our work a trivial correlation was observed for the GLSS T-score and Physical Health T-scores. This was also reflected in the association between Mental Health and the GLSS T-score. This lack of association between general health and life satisfaction may be a reflection of the clinical population in which our work was undertaken. That is, patients presenting with a musculoskeletal complaint may report different outcomes related to global physical and mental health status compared to other clinical presentations. However, this assertion would require additional investigation. Our work also suggests there is a non-existent relationship between pain intensity and life satisfaction, for both acute and chronic duration musculoskeletal presentations. This appears to be the opposite of Australian work by McNamee and Mendolia [38] who reported a strong negative association between chronic pain and life satisfaction. The outcome of the McNamee and Mendolia [38] study may be related to their population including a range of long term health conditions (e.g. cardiovascular disease, respiratory disease), beyond musculoskeletal complaints. Additional research exploring how satisfaction with life intersects with comorbid diseases, health status and pain in populations seeking care for a musculoskeletal complaint would be of benefit.

Health-related behaviours and life satisfaction were also explored in our work, including cigarette smoking, and behaviours related to seeking medical care and buying medicines. Cigarette smoking is recognised as a modifiable health behaviour. In our study, those who reported smoking reported lower life satisfaction however this was not significantly different when compared to non-smokers. This outcome may reflect the younger population in our study compared to the aforementioned works, and the decreasing levels of cigarette smoking in the Australian population [39]. The systematic review by Grant et al. [7] suggested engaging in physical activity and non-smoking were significantly associated with higher life satisfaction. Exploring associations between physical activity and life satisfaction with musculoskeletal health could be valuable, given the links between activity and musculoskeletal complaints [40, 41].

Recent data suggests approximately 28% of Australians delay seeking care from a health professional, and between 4.1%. and 9.7% of Australians report delaying obtaining a prescribed medicine [42]. Whilst the former outcome is consistent with our work, the number of patients who reported delaying buying a medicine was higher. Further, significantly lower GLSS T-scores were exhibited by those who *did not delay* buying medicines or seeking out care from a health professional. This health behaviour may be affected by experiencing a health condition that has a moderate impact on their life, and subsequently reducing their general satisfaction with life ratings. Experiencing a chronic or acute disorder that adversely affects a person’s wellbeing is a motivating factor to reduce or manage symptoms. This outcome suggests these two health behaviours are associated with patients focusing more on their health and accessing care. Previous research suggests health beliefs appear to be independent of life satisfaction [7] but it may be that health behaviours related to accessing care are not. This assertion requires additional investigation. There are many factors that may influence these perceived health beliefs behaviours and pain severity ratings that are beyond the scope of this study, including socioeconomic status [43], and health literacy [44]. It would be helpful to explore these factors in further studies, along with access to health care, participating in recommended national health screening programs, following healthcare advice, and life satisfaction.

### Limitations

The limitations are inherent to consecutive study designs including point-in-time evaluation of a construct(s), and biases such as response, non-response, and social desirability [45] given the measures used in the current work were self-report. These factors may limit the generalisability of the results to other clinical populations and musculoskeletal care environments. In addition, the exploration of the influence of different cultural constructs such as individualist compared to collectivistic countries, did not occur in this study [3]. The lack of Australian data for comparison may limit the ability to make meaningful population comparisons with our data. As quality of life has multiple contributing factors, this is a domain that would need to be explored on an international level, for patients experiencing a variety of conditions apart from musculoskeletal disorders, especially as the data collected for the present study was solely from one manual therapy population.

## Conclusion

Our study suggests that the mean life satisfaction of those presenting for musculoskeletal care at an osteopathy student-led clinic is slightly higher than the American population – the available reference population for the measures used. This study is also the first to describe life satisfaction in a population seeking osteopathy care using a multi-item measure. As such, the data could be used for comparisons with future work into the associations with life satisfaction, mental health, physical health, pain severity and health status in musculoskeletal care.

With respect to health behaviours, demographics and clinical presentation, the present study showed no relationship between pain location, pain severity and life satisfaction. This may in part be accounted for by the younger and less chronic cohort, compared to previous chronic pain literature which did report an association between the two.

Further work to explore the administrative burden of the measure in addition to the relationship of life satisfaction with clinical outcomes is now required and the current study could serve as a basis for this. The GLSS appears to have a degree of utility as a measure of life satisfaction that is readily available to clinicians and researchers.

## Data Availability

The datasets generated and/or analysed during the current study are available on reasonable request from the corresponding author.
